# Mobile device data for the study of miscarriage and its causes

**DOI:** 10.1038/s44294-026-00129-8

**Published:** 2026-03-02

**Authors:** Jenna Nobles, Lindsay Cannon, Sungsik Hwang, Shannon Malloy, Katie Noddin, Allen J. Wilcox

**Affiliations:** 1 University of California, Berkeley, CA USA; 2 University of Wisconsin—Madison, Madison, WI USA; 3 Ovia Health, Boston, MA USA; 4 National Institute of Environmental Health Sciences, Durham, NC USA

**Keywords:** Infertility, Reproductive disorders

## Abstract

Miscarriage is a common pregnancy complication that is difficult to study. Using information on 580,000 US pregnancies from a menstrual- and pregnancy-tracking app, we demonstrate that mobile device data can detect miscarriage and replicate patterns of miscarriage risk found in high-quality Norwegian administrative data and in US clinical cohorts. App data may greatly expand research on miscarriage, including the contributions of “upstream” social, economic, and environmental factors that have eluded study.

Miscarriage—the loss of a clinical pregnancy before 24 weeks of gestation—is common, consequential, and difficult to study in large, diverse populations^1^. Very few countries collect administrative data on miscarriage. Survey data suffer from recall error and desirability bias^2^. Electronic health records in the United States do not have information on most miscarriages because most prenatal clinical care occurs after many miscarriages have already occurred^3^. As a result, scientific research on the causes and consequences of miscarriage is much less well-developed than, for example, research on preterm birth. This study assesses the potential utility of a new source of information on miscarriage—that recorded in mobile device menstrual and pregnancy tracking “apps”—by testing whether user-recorded app data can replicate patterns of miscarriage previously demonstrated in clinical data.

New approaches to the collection of large-scale miscarriage data are welcome, given the difficulty of existing methods. One rigorous approach has been to enroll those trying to become pregnant and follow them prospectively in a “preconception cohort” design^4^. This is the most complete approach because it allows observation of the early weeks of pregnancy, when miscarriage is most common. With rare exceptions^5^, however, the preconception cohort design is prohibitively expensive to conduct in large, representative samples, and is limited to participants who are planning a pregnancy and organized enough to take part in complex longitudinal studies (a selected group). As a result, evidence on the causes of miscarriage has come mostly from clinical studies in limited locations among non-representative persons. Such evidence cannot speak to the causes of miscarriage that originate in aspects of families, neighborhoods, or communities. Available studies are largely limited to information about causes attributed to pregnant people themselves, such as diet, behavior, or health conditions. These personal attributes and behaviors dominate the known risk factors for miscarriage^6^.

Scientific emphasis on individual-level causes of miscarriage implicitly or explicitly attributes the “cause” of miscarriage to the person carrying the pregnancy, versus a larger set of biological, environmental, and societal causes^7^. There is strong theoretical support for the relevance of “upstream” causes^8,9^—e.g., poverty, violence, housing conditions, air pollution, state and federal health policies—that have known consequences for other dimensions of pregnancy health^10,11^. To date, rigorous empirical research on whether and how these exposures contribute to miscarriage is extremely limited, largely because there are few data resources to analyze them, and even fewer that support quasi-experimental designs with causal interpretation.

Mobile device “apps” that allow people to track menstrual cycles and pregnancies are well-suited for the collection of health information at the scale of populations, particularly on under-studied dimensions of reproductive health^12–16^. Mobile device apps could facilitate unprecedented collection of data on variation in miscarriage risk over time and place, by providing low-cost data-spanning pregnancy cohorts that place a low burden on participants. Data indexed in time and place would facilitate the linkage of early pregnancy outcomes to variation in contextual exposures, and would make it possible to identify a wide range of upstream predictors of miscarriage risk. Such data would also facilitate the use of quasi-experimental design for causal inference; an approach now widely used to study the effects of upstream social, economic, and environmental determinants on other pregnancy outcomes, including preterm birth^17–19^.

Before research on miscarriage can be developed using data from mobile device health tracking apps, it is necessary to document the utility and validity of such data. We assess mobile device data on miscarriage by analyzing information from a free-to-consumer fertility app used by several million U.S. residents between 2016-2022 to record menstrual cycles and pregnancies. We examine two patterns of miscarriage risk that have been previously documented in clinical research in the U.S. and demonstrated in high-quality registry data collected in Norway. In Norway, all miscarriages that include clinical visits are documented in health records. Researchers have studied these miscarriages beginning at approximately 6 weeks of gestation (i.e., the start of the 7^th^ week of pregnancy)^20^. Prenatal care is free and easily available in Norway, and nearly all pregnancies are captured in clinical records by the end of the first trimester^20,21^.

Using the app data, we assess miscarriage risk by maternal age and prior history of miscarriage, two risk factors thought to be largely biological in origin and similar across high-income populations^1^. We then compare the patterns in the app data with those previously established in the Norwegian registry data. We find that the results using U.S. app data closely replicate patterns in pregnancy loss demonstrated in these other data sources, even though the app data are self-reported through a mobile device and not captured for clinical purposes. By replicating these two patterns, we are persuaded that app data can be an important resource to advance miscarriage research – particularly for the study of miscarriage in large, heterogeneous, or low-income populations that are systematically underrepresented in clinical research. In addition, app data contains information across cohorts of pregnancies that span years and geographic areas. Such digital sources could provide information on understudied but potentially relevant societal, environmental, and policy drivers of miscarriage.

Figure 1 plots miscarriage risk by the age of the pregnant person among pregnancies that survive through 6 weeks of gestation. Data for the U.S. mobile device data are shown in orange, and the Norwegian registry data in blue (previously described in Magnus et al.^20^). The pattern of age-specific risk is strikingly similar for the two groups. In the digital data, risk is slightly elevated at the youngest ages, reaching its lowest value around ages 23-26, and then increasing beyond age 27. This “J-shaped” curve in the U.S. digital data closely matches the pattern in the Norwegian registry data, with a nearly identical slope over maternal age. Similar patterns of elevated risk in adolescence and at advanced maternal age have also been described in multiple cohorts in the U.S^22,23^. Supplementary Fig. 1 adds a third line with older estimates recorded from New York City clinical samples (previously described in Wilcox 2010^1^). These also follow a highly similar shape over maternal age.

**Fig. 1:**
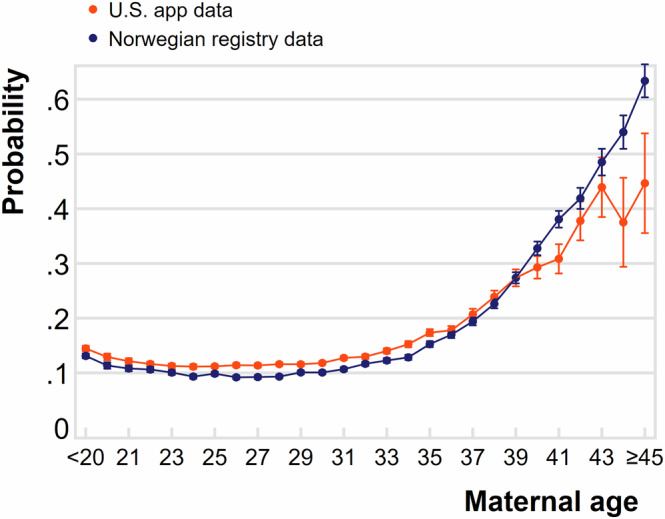
Probability of pregnancy ending in miscarriage by maternal age (years), in the United States app data and Norwegian registry data. Pregnancies recorded in a large U.S. app between 2016–2021 that survive through 6 weeks of gestation are shown in orange (authors’ original analysis). Pregnancies measured in Norwegian registry data from 2009 to 2013 in navy; Norwegian data have been generously provided by Maria Magnus^20^. Probabilities and 95% confidence intervals are depicted. Probabilities for U.S. app data estimated via maternal age-specific lifetables using a sample of 589,413 pregnancies. App estimates weighted with post-stratification weights. Norwegian registry estimates are proportions calculated from the full census of Norwegian pregnancies.

Figure 2 plots the age-adjusted miscarriage risks by previous miscarriage experience, for U.S. app data (in orange) and Norwegian data (in blue). Miscarriage risk increases with the reported number of previous miscarriages. For app users, the odds ratio of miscarriage after one previous miscarriage relative to those with none is 1.50 (CI: 1.08–2.08), after two previous miscarriages, 1.93 (CI: 1.52–2.45), and after 3 or more previous miscarriages, 3.23 (CI: 2.06–5.06). This dose relationship is similar for the two data sources.

**Fig. 2:**
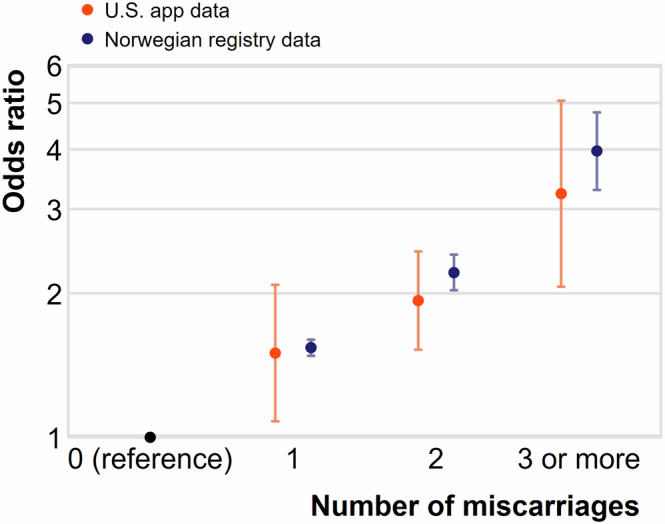
Adjusted odds ratio of pregnancy ending in miscarriage, by experience of miscarriage in previous pregnancies, in the United States app data and Norwegian registry data. Odds ratios and 95% confidence intervals estimated on a sample of 77,527 pregnancies from US residents recorded in an app between 2016–2021 that survive through 6 weeks of gestation (orange, authors’ original analysis) and pregnancies measured in Norwegian registry data from 2009–2013 (dark blue, previously reported in Magnus et al. (2019)). Odds ratios in both data sources are adjusted for maternal age (years). App data estimates are weighted with post-stratification weights.

Building on this replication, digital data with information on date and region may be particularly useful for a rigorous study of sources of variation in miscarriage risk, including upstream factors^24^. To date, the effects of potentially important social and environmental exposures on pregnancy loss are largely unexplored. There has been a transition from focus on individual-level risk factors to institutional and societal-level risk factors for other pregnancy outcomes, including maternal morbidity and newborn health. This shift has promoted the development of research and policy to support healthier pregnancies and improve infant survival^10,11,25^. A similar expansion of research on miscarriage has not previously been possible. Digital data provides one way to support this advance.

One concern about data derived from mobile apps has been that the data could be used to surveil people for punitive purposes^26^. Our analyses use de-identified data that show miscarriage probabilities in the aggregate a strategy that can be applied to other miscarriage research without jeopardizing the privacy concerns of individual users. This would support scientific and public understanding of miscarriage as a population health outcome one shaped by a wider set of exposures than the behaviors and characteristics of pregnant individuals. To this end, research with protected, de-identified mobile device data may further *challenge* the logic of individual attribution and surveillance.

Digital data has its own limitations. Pregnancy and miscarriage are based on respondent reports rather than biological samples. It is not possible to identify chromosomal abnormalities among the miscarriages, which are thought to cause about half of miscarriages^27^. We estimated gestational age from respondent reports of last menstrual periods and pregnancy due dates. The imprecision of such dates can cause misclassification of pregnancies that spontaneously abort before and after week 6, and therefore misclassification of pregnancies in the analytic sample. We note, however, that the results are not changed by shifting the gestational window to weeks 8-24 (see online Supplement).

The users of apps are themselves not necessarily representative of the whole population, and the nature of sample selectivity may change over time. We have used a weighting method to address sample selectivity on several dimensions. This method cannot adjust for unmeasured attributes that are uncorrelated with the attributes used to create the weights. Nevertheless, the general weighting approach we employ could be expanded to capture more complex forms of sample selectivity whenever these can be adequately measured in both sample and population data.

The tests shown here are encouraging. They cannot speak to the validity of app data for research on upstream causes of miscarriage; however, they do support the pursuit of such research. Given the difficulty of otherwise studying miscarriage in large, diverse samples, mobile device data are an imperfect but promising way forward.

## Methods

### Data

We use data from menstruating U.S. residents who recorded information in Ovia Fertility, a free mobile app available on Android and iOS platforms in English and Spanish that allows users to document multiple dimensions of menstruation and attempts at pregnancy, including physical symptoms, such as bleeding, breast tenderness, headaches, nausea; intercourse; the use of medications; and the results of pregnancy tests^15^. Users can also elect to answer a set of survey questions about medical history, demographics, behaviors, and partner and family characteristics. Following a pregnancy report, respondents are invited to use a linked app, Ovia Pregnancy (similarly available at no cost in both English and Spanish), that allows users to document pregnancy symptoms, health-care visits, and information about the delivery and infant.

At sign-up, users of Ovia Health apps agree to Ovia Health’s Privacy Policy, which permits Ovia Health to create de-identified data for research. Significant protections are in place to ensure user privacy. This research was approved by the University of Wisconsin-Madison Institutional Review Board (registration #IRB00003739). The authors have complied with all relevant ethical regulations, including the Declaration of Helsinki.

We prospectively followed 589,413 people aged 18-49 years who met the following criteria: (1) they reported trying to conceive for less than six months at the time of enrollment (minimizing selection of those with low fecundity); (2) they used the app more than five times a month prior to reporting the positive pregnancy test (associated with better data quality^12^) and (3) they reported a positive pregnancy test between January 1, 2016 and February 15, 2022. When weighted as described below, the overall miscarriage probability in the sample is 21% and the mean age of the sample is 32.6 years. This overall miscarriage probability aligns closely with estimates reported in other U.S. samples (22% in Li et al.^28^; 19% in Yland et al.^29^; 23% in Wesselink et al.^30^).

### Measurement

To conduct this study, we use information on recorded menstrual cycles, on the timing and outcome of pregnancy tests, on pregnancy outcomes, and on people’s age in years, zip code of residence, and previous miscarriages.

We define miscarriage as the reported resumption of menstrual cycles after a pregnancy lasting less than 24 weeks. The start of gestation is indicated by the beginning of the last menstrual period. It is possible that this will misclassify some surviving pregnancies as miscarriages. However, misclassification appears to be rare: among the 86,550 pregnancies during which users report the resumption of menstrual cycles, only 640 users (less than 1%) subsequently report a live birth from the pregnancy. Results are robust to reclassifying these 640 pregnancies as live births. The use of other definitions of miscarriage—including termination prior to 20 weeks or 28 weeks of gestation—does not substantively change the results. Pregnancy loss after week 20 of gestation is rare, occurring in less than 1% of pregnancies^1^.

We minimize the probability of inadvertently including induced abortion among miscarriages by limiting the sample to people who reported they were “trying to conceive” at the time they signed up for the app. Data from the Guttmacher Institute’s 2014 Abortion Patient Survey indicate that, at maximum, 3% of induced abortion patients in the U.S. were trying to conceive when they became pregnant. Between 10-15% of all pregnancies ended in induced abortion in 2014^31^ (see online Supplement), and about half of pregnancies were intended; as a result, approximately 1% of intended pregnancies end in induced abortion (via Bayes theorem; see online Supplement for this calculation). We conclude that the contribution of misclassified induced abortions to the results shown here is likely negligible.

### Sample attrition

Among the 589,413 people in the analytic sample, 57,819 (9.8%) attrited before week 24 of gestation. In the online Supplement, we plot attrition by maternal age and previous miscarriage experience. Attrition is not correlated with either measure and thus does not affect the relationships shown in Figs. 1 or 2.

### Analysis

We conducted two analyses presented in the Norwegian registry data. One is the risk of miscarriage by the age of the pregnant person, and the other is the risk by the number of previous miscarriages. The estimates reported here include miscarriages beginning in week 6 of gestation^20^. Unlike the U.S., prenatal care in Norway is nearly universal^21^. To replicate these tests, we limit the sample of pregnancies in the app data to those that survive through at least 6 weeks of gestation. Results are not substantially changed when limiting the app sample to those that survive through at least 8 weeks of gestation (see online Supplement).

We estimate the probability of miscarriage by maternal age, measured in single years. We address right-censoring in the data by using lifetables^32^ generated for each year of maternal age to estimate cumulative probabilities of miscarriage between week 7-24 of gestation, among pregnancies that survive through week 6 of gestation.

To improve population representativeness of the estimates, we generate post-stratification weights at the level of the zip code, which is a well-established predictor of health in the U.S^33^. and is well-measured in the app data (see online Supplement for details). Post-stratification weights are constructed by relating the distribution of observed characteristics in an analytic sample to those same characteristics in a population. We generate weights separately for the samples in Figs. 1 and 2. The weighted samples thus match the distribution of reproductive-age women across zip codes jointly characterized by racial/ethnic composition, poverty, and population density. We also present unweighted results in the supplementary appendix; the results are not sensitive to the inclusion of weights.

All app users provide their age in years. App users can choose to answer survey questions about their previous fertility experiences, among other dimensions of health and welfare. 13% of those reporting a positive pregnancy test also provided information about previous miscarriages (*N* = 77,527 pregnancies). We use logistic regression to estimate the odds of miscarriage by prior miscarriage experience (0, 1, 2, 3+ prior miscarriages), adjusting flexibly for maternal age (in years) with single-year age fixed-effects. Post-stratification weights generated for this subsample of pregnancies help address nonrandom selection into the provision of additional information about fertility history. We also compared measured characteristics for the 13% of respondents who provided additional information about miscarriage and the 87% who did not (see Supplemental Appendix). The two samples are highly similar. A sensitivity analysis adjusting for differential non-response produces equivalent results (Supplemental Appendix).

## Supplementary information

Supplementary Material

## Data Availability

To protect user privacy and confidentiality, restrictions apply to the availability of the data, and so they are not publicly released. Data are available from Ovia Health upon reasonable request and with necessary data protections, reviews, and agreements.

## Code availability

Code to generate the Figures in this study is at https://github.com/jen-nej/miscarriage/.
