# Supplementary material for: Mobile device data for the study of miscarriage and its causes
Source: NPJ Womens Health. 2026 Mar 2;4(1):11. doi: 10.1038/s44294-026-00129-8 (PMC12953145; doi:10.1038/s44294-026-00129-8)
Supplement: Supplementary file 1 — Supplementary Material [file 44294_2026_129_MOESM1_ESM.pdf]

Supplementary Material:
Mobile Device Data for the Study of Miscarriage and its Causes

Jenna Nobles[1,2], Lindsay Cannon[2], Sungsik Hwang[2], Shannon Malloy[3], Katie Noddin[3], Allen J. Wilcox[4]

[1]University of California, Berkeley
[2]University of Wisconsin, Madison
[3]Ovia Health
[4]National Institute for Environmental Health Sciences

Contents:

1. Post-stratification weights
2. Proportion of intended pregnancies that end in abortion in the U.S.
3. Additional evidence from U.S. clinical cohorts
4. Miscarriage estimates without application of post-stratification weights
5. Similarity of age and area-level characteristics among users with and without information on previous miscarriage experience.
6. Estimates in which app-reported pregnancies are limited to pregnancies that survive to gestation week 8.
7. Estimates among people in lower SES zipcodes and without college degrees.
8. Estimates excluding the COVID-19 period: 2020-2022
9. Similarity of maternal age and previous miscarriage experience among sample that is observed through week 24 of gestation and sample that attrites before week 24.

1. Post-stratification weights.

Post stratification weights are used to match the distribution of a sample over a matrix of characteristics to the distribution of a population over the same matrix of features.[1] Because zipcode is highly predictive of health and wellbeing in the U.S. and is well-measured in these data, we use post-stratification weights that leverage characteristics measured at the zipcode level. We cross-classify U.S. zipcodes by race/ethnic composition (majority non-Hispanic Black (>50%), majority Hispanic (>50%), majority non-Hispanic white (50-90%), predominantly non-Hispanic white (>90%), and all others), poverty level (0-8%, 8-15%, 15-30%, 30-100%), and urban status (metropolitan, micropolitan, small town, rural), and missing Census criteria using data from the U.S. Census Bureau, producing 81 zipcode classifications (5x4x4+1). We estimate the

fraction of U.S. reproductive-age women living in each of those types of zipcodes and the fraction of women in the analytic samples in each of those types of zipcodes. The weights are the ratio of those two distributions.

[1]Little RJ. Post-stratification: a modeler's perspective. Journal of the American Statistical Association. 1993 Sep 1;88(423):1001-12.

2. Proportion of intended pregnancies that end in abortion in the U.S.

The 2014 Abortion Patient Survey[2] is the most recent public data source that facilitates the study of pregnancy intendedness among a population-representative sample of abortion patients in the U.S. In the 2014 data, at most 3% of abortion patients intended the pregnancy at the time of conception. The proportion of all pregnancies that ended in abortion in 2014 fell in the range 10.3-11.6%, calculated using a conservatively wide range of 20%-30% of pregnancies ending in pregnancy loss (including subclinical early pregnancy loss, miscarriage, and stillbirth). The proportion of all pregnancies that were intended at conception was 45%, estimated using a standard approach that estimates the fraction of pregnancies intended at conception, P_i / P, as a function of the proportion of abortions intended at conception, A_i / P, and of live births intended at conception, B_i / P, in the same year (eq. 1, Kost, Zolna, and Murro 2023[3]). The approach, in effect, assumes that pregnancy losses have slightly lower rates of intendedness than live births. By Bayes' Theorem (eq. 2), the proportion of intended pregnancies ending in abortion was less than 1%, and specifically took the range of values from 0.7%-0.8%. Though more recent Abortion Patient Survey data are not available, we used the 2008 APS files to generate an estimate of 1.3%-1.4% of pregnancies that were intended at conception ending in abortion in 2008. We conclude that this outcome—abortion among intended pregnancies—is rare and not a meaningful source of bias in the estimates shown.

1. $P_i/P = 1.2\ (B_i / P) + 1.1(A_i/P)$
2. $Pr\ (A|B) = (Pr(B|A)\ Pr(A))\ /\ Pr(B)$

[2]Abortion Patient Survey data files: https://www.openicpsr.org/openicpsr/project/163962/version/V1/view

[3]Kost K, Zolna M, Murro R. Pregnancies in the United States by desire for pregnancy: estimates for 2009, 2011, 2013, and 2015. Demography. 2023 Jun 1;60(3):837-63.

3. Additional evidence from U.S. clinical cohorts.

Supplementary Figure 1 includes estimates from clinical data collected in New York City, represented in Wilcox (2010). These are depicted in black, with open circles, and closely align with the level and shape as the two other series depicted.

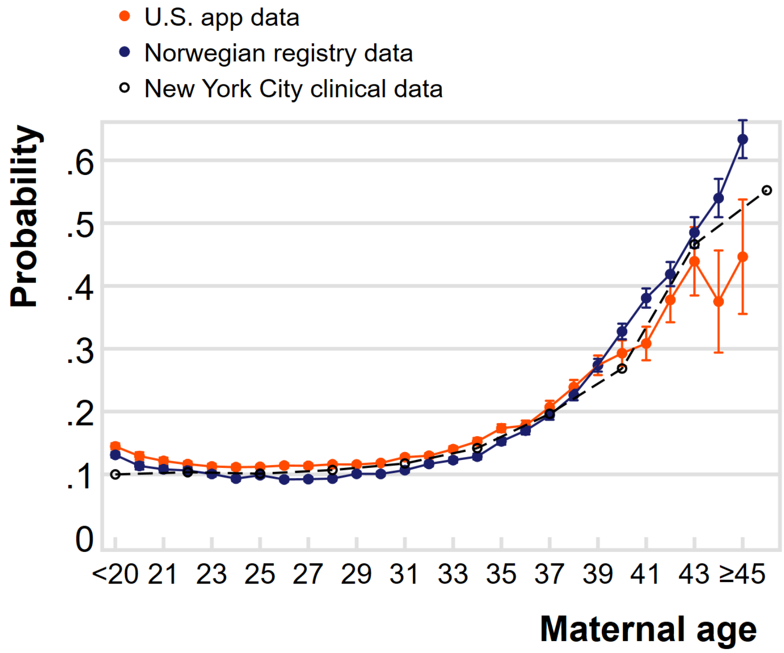

Supplementary Figure 1. Probability of pregnancy ending in miscarriage by maternal age, in United States app data and Norwegian registry data. Pregnancies recorded in a large U.S. app between 2016-2021 that survive through 6 weeks gestation in orange. Pregnancies measured in Norwegian registry data from 2009-2013 in navy. Pregnancies measured in New York City clinical cohorts in black. Probabilities and 95% confidence intervals (for U.S. app data and Norwegian registry data) are depicted. App estimates weighted with post-stratification weights.

4. Miscarriage estimates without application of post-stratification weights

Supplementary Figures 2 and 3 display estimates of miscarriage risk shown in Figures 1 & 2 in the main manuscript, respectively, without the use of post-stratification weights.

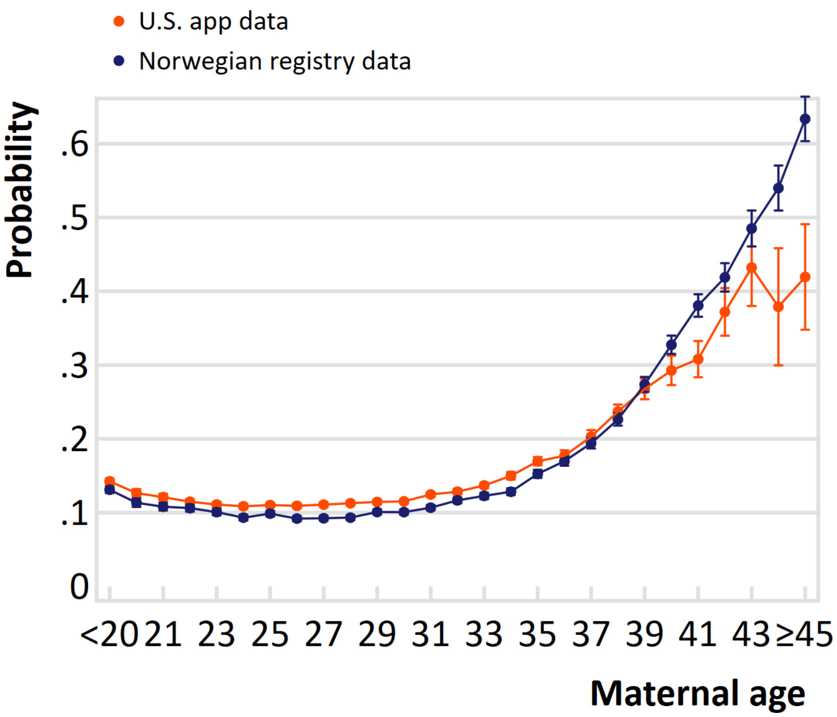

Supplementary Figure 2. Probability of pregnancy ending in miscarriage by maternal age, in United States app data and Norwegian registry data, estimates not weighted. Pregnancies recorded in a large U.S. app between 2016-2021 that survive through 6 weeks gestation in orange. Pregnancies measured in Norwegian registry data from 2009-2013 in navy. Probabilities and 95% confidence intervals are depicted. App estimates are unweighted.

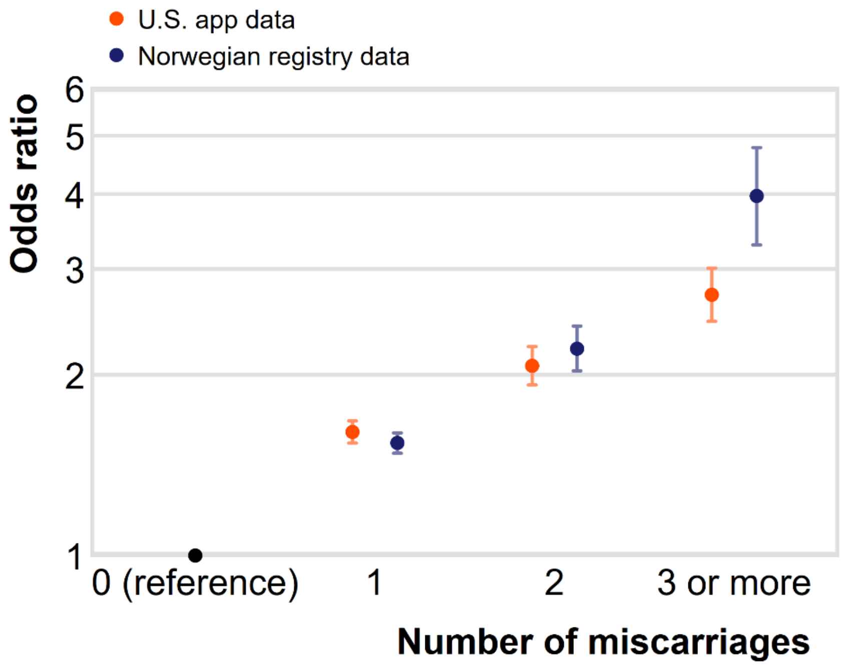

Supplementary Figure 3. Adjusted odds ratio of pregnancy ending in miscarriage, by experience of miscarriage in previous pregnancies, in United States app data and Norwegian registry data, estimates unweighted. Odds ratios and 95% confidence intervals estimated on sample of 77,527 pregnancies from US residents recorded in an app between 2016-2021 that survive through 6 weeks gestation (orange) and pregnancies measured in Norwegian registry data from 2009-2013 (navy), previously reported in Magnus et al. 2019. Odds ratios in both data sources are adjusted for maternal age (years). App data estimates are not weighted.

5. Similarity of age and area-level characteristics among users with and without information on previous miscarriage experience.

In Supplementary Figure 4, below, we plot the distribution of individual zipcode level poverty (in percentage points), racial/ethnic composition (in four categories), and population density (in four categories using the U.S. Census Bureau classification). We plot these distributions for the sample of people who provide information on previous miscarriage (plotted in orange) and for the sample of users who do not provide information on previous miscarriage (plotted in navy). The sample in navy contributes information to Figure 1 but not Figure 2 in the main text.

We observe that the samples are highly similar on these characteristics. Users who do not provide information on miscarriage history live in zip codes with slightly higher poverty rates but the difference is small: < 2 percentage point difference in the probability of living in a zip code with a >15% poverty rate between people with and without information on previous miscarriage history. Nonresponse about miscarriage history does not appear to be meaningfully selective on measured sociodemographic characteristics.

As a further test that these differences are not large enough to affect the conclusions of this study, we re-analyze the data used to generate the estimates in Figure 2, using inverse probability weights (Wooldridge 2007[4]). These weights adjust for differential nonresponse by the same measured sociodemographic characteristics: age (in years, parameterized with both a linear and quadratic term), zip code level poverty (in percentage points), racial/ethnic composition (in four categories), and population density (in four categories using the U.S. Census Bureau classification). The results are plotted in Supplementary Figure 5, below. The estimates are unchanged with and without this adjustment for differential item non-response.

[4]Wooldridge, J. M. (2007). Inverse probability weighted estimation for general missing data problems. *Journal of econometrics*, *141*(2), 1281-1301.

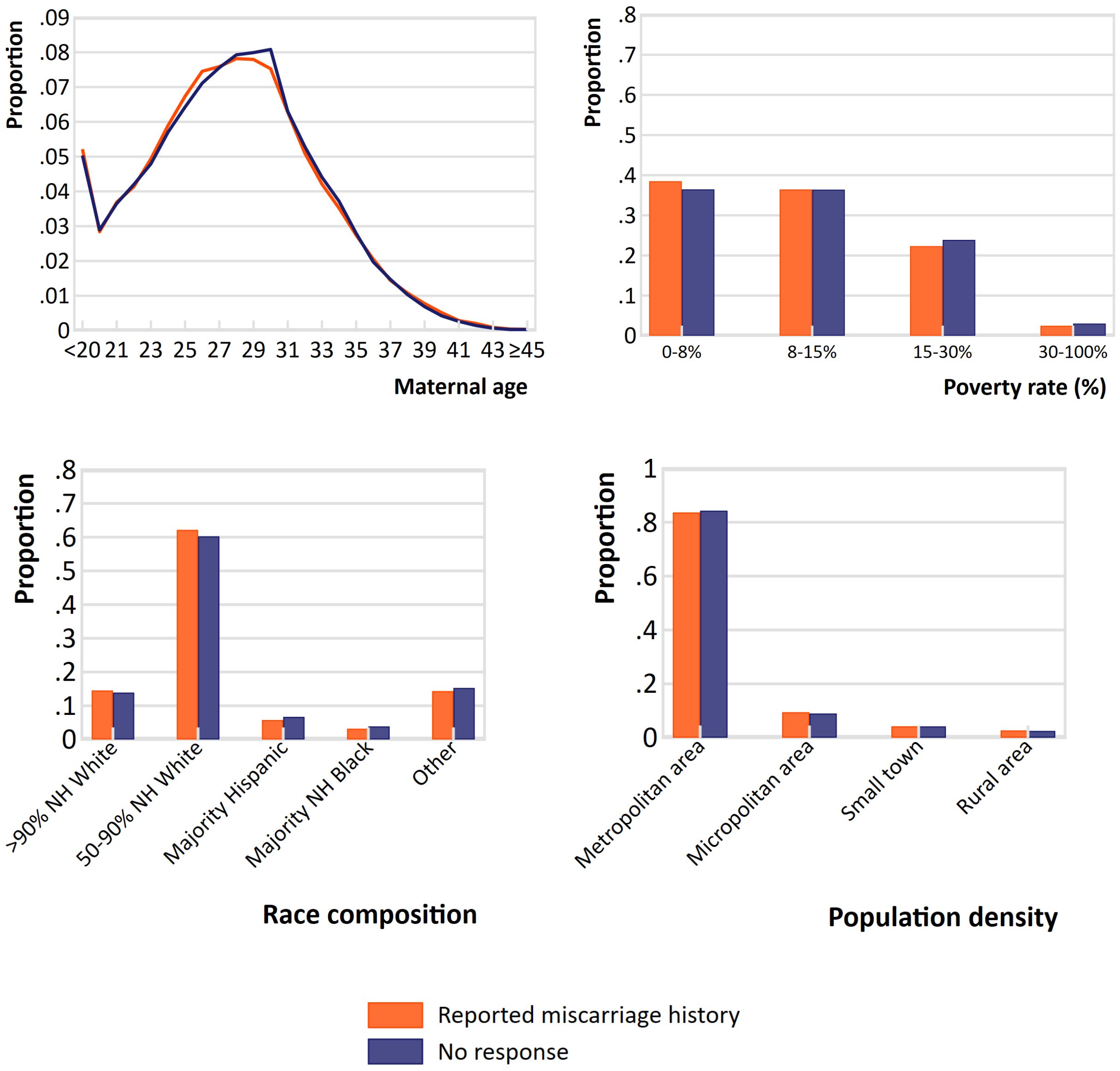

Supplementary Figure 4. Comparison of the characteristics in the U.S. app sample among users who provide information about previous miscarriage history, including no previous miscarriages (orange) and the U.S. app sample among users who do not provide information about previous miscarriage (navy).

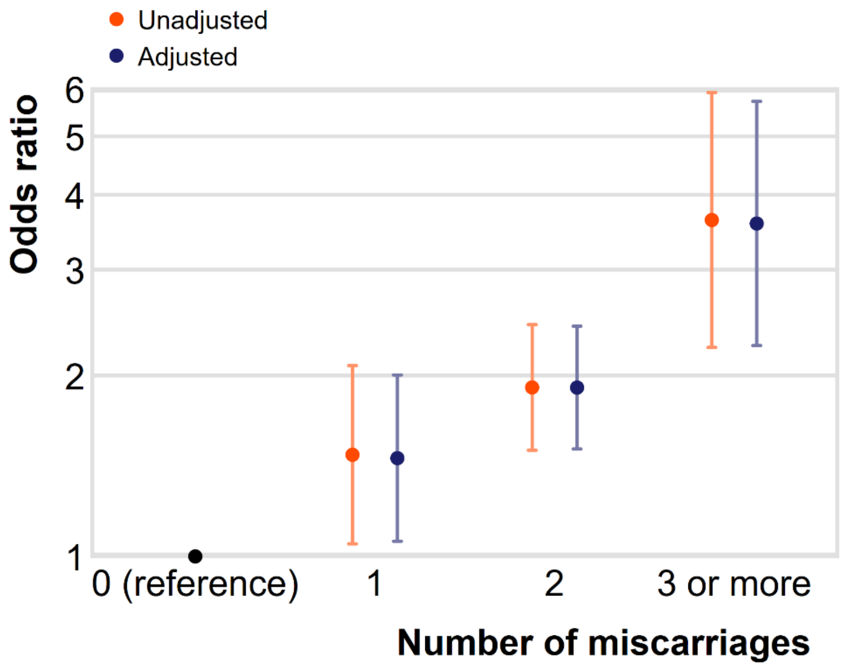

Supplementary Figure 5. Age-adjusted odds ratio of pregnancy ending in miscarriage, by experience of miscarriage in previous pregnancies, in United States app data and Norwegian registry data, unadjusted (orange) and adjusted (navy) for differential item nonresponse by sociodemographic characteristics. Odds ratios and 95% confidence intervals estimated on sample of 77,527 pregnancies from US residents recorded in an app between 2016-2021 that survive through 6 weeks gestation. Odds ratios are adjusted for maternal age (years). Both estimates are weighted with post-stratification weights.

6. Estimates in which app-reported pregnancies are limited to pregnancies that survive to gestation week 8.

Miscarriages in Norwegian health record data can be observed as early as 6 weeks into gestation. To be observed, people must report the miscarriage to their doctor or seek medical attention. To the extent that this is more likely to happen at, e.g., week 9 of pregnancy than at week 6, it is possible that the Norwegian register data has a smaller fraction of pregnancies that terminate at week 6 than at week 9. In the primary analysis, the app data include all miscarriages that occur at week 6, rather than a fraction of them. To test the robustness of the comparison between the Norwegian register data and the app data to potential missingness in the Norwegian data, we re-estimate Figure 1 after limiting the app sample to pregnancies that survive to week 8 of pregnancy. These are plotted in Supplementary Figure 6, below. the conclusions are unchanged. We find a similar J-curve along maternal age closely matching the shape of that plotted in Magnus et al. 2019. At each age, the probabilities of miscarriage are slightly lower than in the original estimates, which is expected given the higher risk of miscarriage before week 8 versus after week 8 of pregnancy.

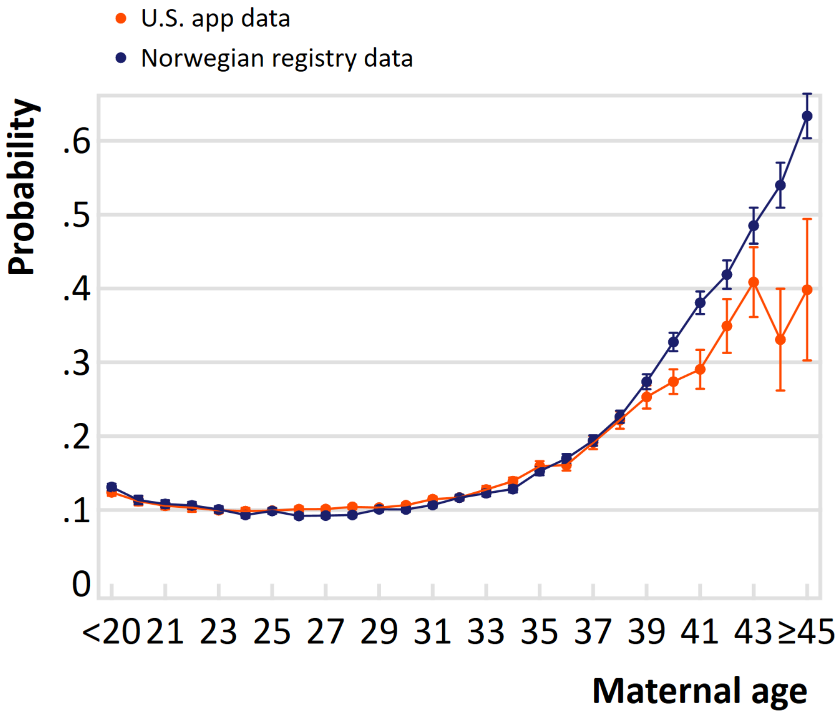

Supplementary Figure 6. Probability of miscarriage by maternal age (in years), Norwegian registry data (in navy) and U.S. app data limited to pregnancies surviving to 8 weeks gestation (in orange). Pregnancies recorded in a large U.S. app

between 2016-2021 that survive through 8 weeks gestation in orange. Pregnancies measured in Norwegian registry data from 2009-2013 in navy. Probabilities and 95% confidence intervals are depicted. App estimates weighted with post-stratification weights.

7. Estimates among people in higher-poverty areas.

One concern is that the similarity of findings between app users and people who do not use apps is due to the comparatively higher socioeconomic status (SES) of the sample. Below, we plot Figure 1 and 2 for people in the U.S., excluding people living in higher SES zip codes—places where fewer than 8% of residents in the zip code live below the U.S. Federal poverty guideline. The results are unchanged. We conclude that the similarity in patterns across the two samples is not solely because of higher-SES individuals.

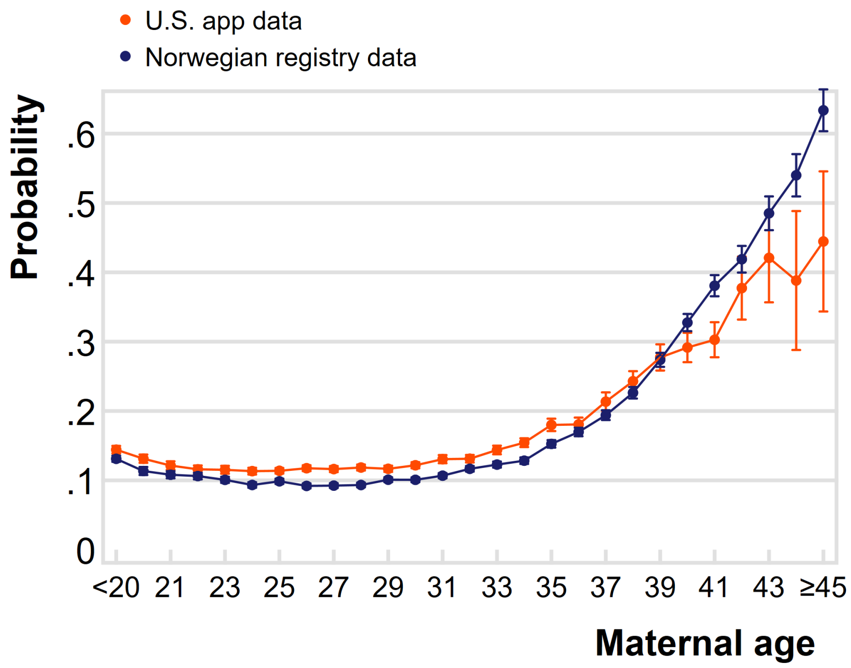

Supplementary Figure 7. Probability of miscarriage by maternal age (in years), Norwegian registry data (in navy) and U.S. app data limited to users living in higher-poverty areas (in orange). Pregnancies recorded in an U.S. app between 2016-2021 that survive through 6 weeks gestation in orange. Pregnancies measured in Norwegian registry data from 2009-2013 in navy. Probabilities and 95% confidence intervals are depicted. App estimates weighted with post-stratification weights.

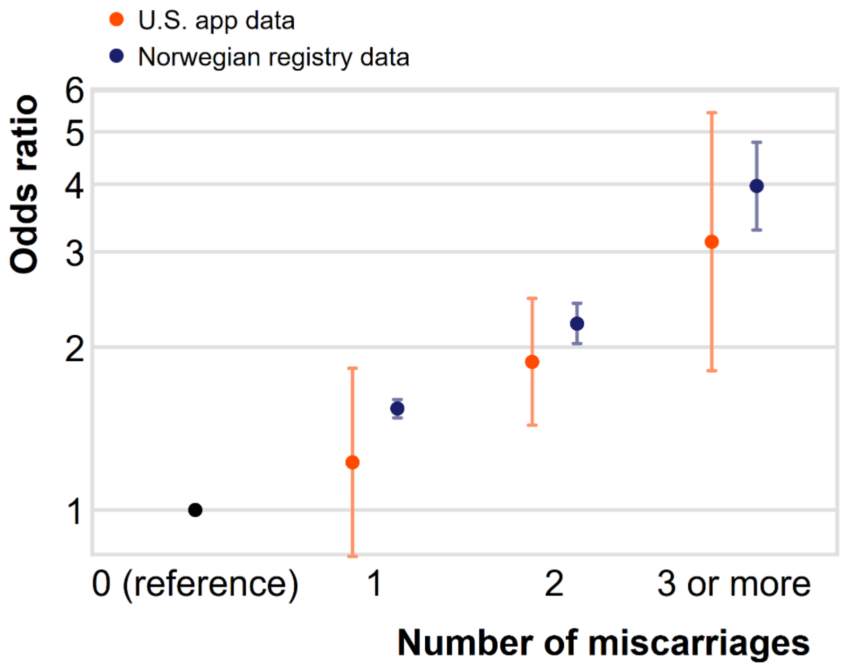

Supplementary Figure 8. Age-adjusted odds ratio of pregnancy ending in miscarriage, by experience of miscarriage in previous pregnancies, in Norwegian registry data (in navy) and U.S. app data limited to users living in higher-poverty areas (in orange). Odds ratios and 95% confidence intervals estimated on pregnancies recorded in an app by US residents between 2016-2021 that survive through 6 weeks gestation. Odds ratios in both data sources are adjusted for maternal age (years). App estimates weighted with post-stratification weights.

8. Estimates excluding the COVID-19 period: 2020-2021

The results hold after excluding the years during the COVID-19 pandemic.

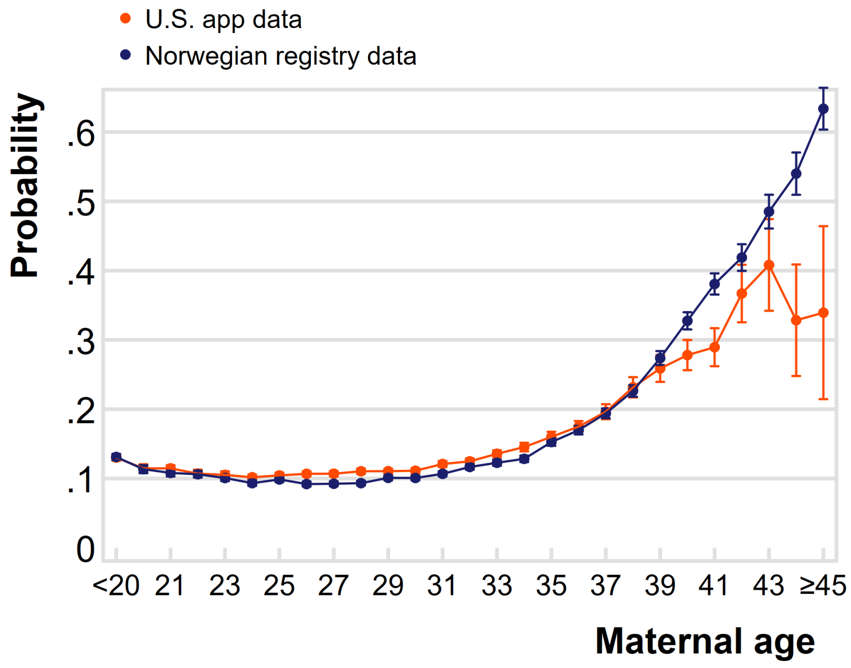

Supplementary Figure 9. Probability of miscarriage by maternal age (in years), Norwegian registry data (in navy) and U.S. app data excluding the COVID-19 period (in orange). Pregnancies recorded in a U.S. app between 2016-2019 that survive through 6 weeks gestation in orange. Pregnancies measured in Norwegian registry data from 2009-2013 in navy. Probabilities and 95% confidence intervals are depicted. App estimates weighted with post-stratification weights.

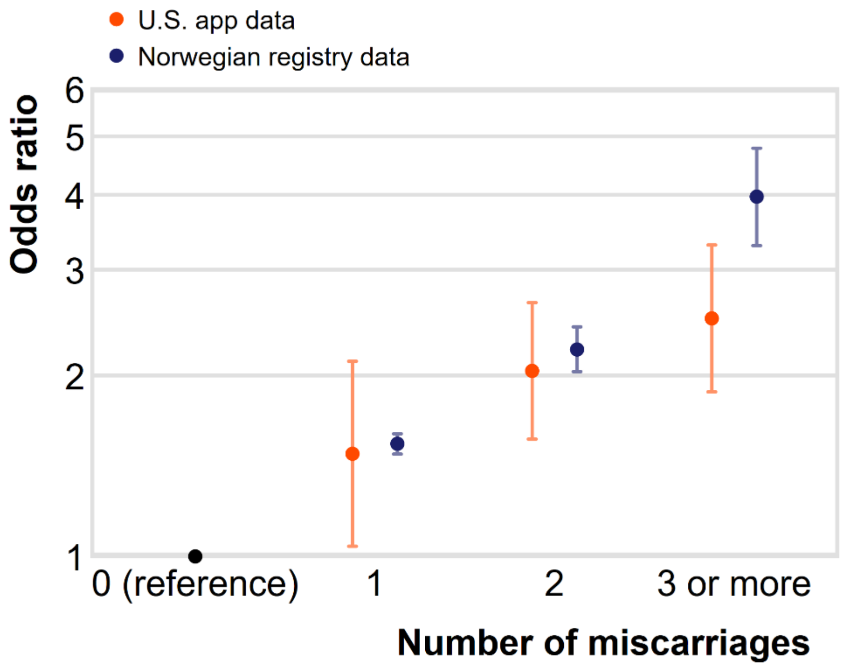

Supplementary Figure 10. Age-adjusted odds ratio of pregnancy ending in miscarriage, by experience of miscarriage in previous pregnancies, in Norwegian registry data (in navy) and U.S. app data excluding the COVID-19 period (in orange). Odds ratios and 95% confidence intervals estimated on pregnancies recorded in an app by US residents between 2016-2019 that survive through 6 weeks gestation. Odds ratios in both data sources are adjusted for maternal age (years). App estimates weighted with post-stratification weights.

9. Similarity of maternal age and previous miscarriage experience among sample that is observed through week 24 of gestation and sample that attrites before week 24.

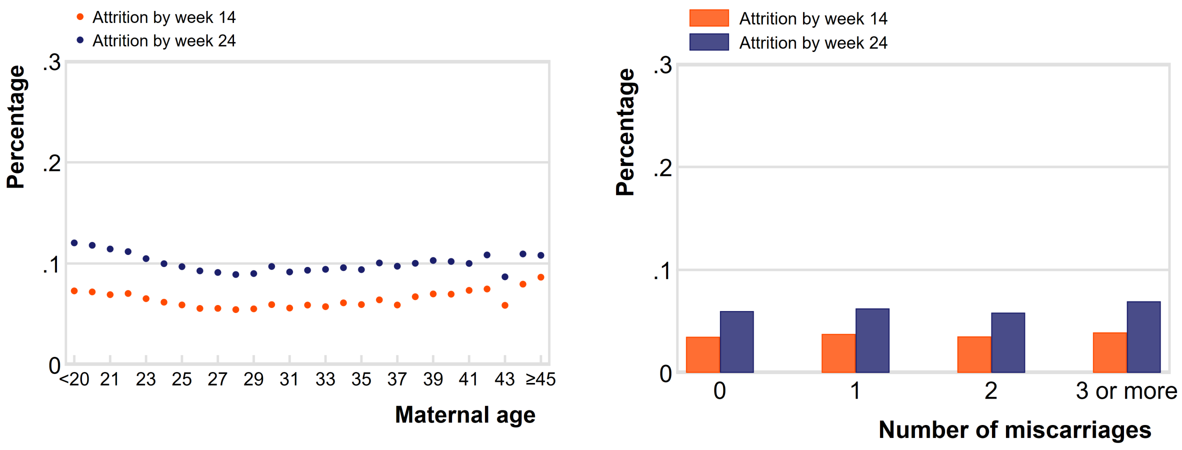

Supplementary Figure 11. The percentage of sample attrition before week 14 (orange) and week 24 (navy) by maternal age and previous number of miscarriages in the U.S. app data.
